# COVID-19 induced thrombotic thrombocytopenic purpura in a patient with systemic lupus erythematosus: A rare case report

**DOI:** 10.1097/MD.0000000000040992

**Published:** 2024-12-27

**Authors:** Ou Gao, Yinghua Chen, Honglang Xie

**Affiliations:** a National Clinical Research Center of Kidney Diseases, Jinling Hospital, Nanjing University, Nanjing, China.

**Keywords:** COVID-19, lupus nephritis, SARS-CoV-2, systemic lupus erythematosus, thrombotic thrombocytopenic purpura

## Abstract

**Rationale:**

Thrombotic thrombocytopenic purpura (TTP) is a severe and rare disease, and its complexity increases in the presence of underlying autoimmune disease and COVID-19 infection, making differential diagnosis and treatment more challenging.

**Patient’s concerns:**

A 43-year-old patient presented with high fever, intermittent cough, and tea-colored urine.

**Diagnoses:**

The patient had a long-term history of systemic lupus erythematosus (SLE) and lupus nephritis (LN). The nasopharyngeal swab confirmed the diagnosis of COVID-19 by RT-PCR, and plasma ADAMTS-13 activity was completely deficient (0%). It was considered that COVID-19 infection occurred on the basis of SLE disease and was then complicated with TTP.

**Interventions:**

The patient was successfully treated with plasma exchange, followed by a combination of biologics and immunosuppressants.

**Outcomes:**

After 1 year of follow-up, the patient had completely recovered from COVID-19 infection and TTP, meeting the cure criterion. In addition, the LN was in remission, with an SLEDAI-2K score of 0, indicating a low disease activity state.

**Lessons:**

This article indicates that the patient suffers from both long-standing underlying diseases and the sudden occurrence of SARS-CoV-2 infection, which complicates the determination of the etiology and diagnosis of TTP. Consequently, after thorough analysis of the disease progression, clinical manifestations, laboratory results, and treatment outcomes, it was primarily concluded that COVID-19 was the catalyst for the onset of TTP in this patient.

## 1. Introduction

Thrombotic thrombocytopenic purpura (TTP) is a disorder of endothelial dysfunction characterized by autoantibodies inhibiting the metalloprotease ADAMTS-13. This condition predominantly manifests as microangiopathic hemolytic anemia (MAHA), thrombocytopenia, neuropsychiatric symptoms, fever, and renal failure. While the exact causal relationship requires further investigation, both COVID-19 infection and systemic lupus erythematosus (SLE) have been implicated in the potential induction of TTP through mechanisms such as direct endothelial damage, production of anti-ADAMTS-13 antibodies, and autoimmune responses. This article details a case of a patient with a 15-year history of SLE and lupus nephritis (LN), who experienced an acute onset of TTP following a COVID-19 infection. The patient’s clinical symptoms improved following the administration of methylprednisolone pulse therapy, plasma exchange (PE), and rituximab.

## 2. Case description

The patient is a 43-year-old male with a history of LN class IV, confirmed by renal biopsy in 2008, and has been on long-term immunosuppressive treatment with methylprednisolone and mycophenolate mofetil. This treatment regimen successfully normalized his serum creatinine levels, reduced urinary protein, and maintained SLE in remission.

On May 3, 2023, the patient developed a fever of 38.2°C, intermittent cough, general fatigue, and tea-colored urine. An emergency nasopharyngeal swab tested positive for SARS-CoV-2 using a RT-PCR test. Initial blood work revealed a platelet count of 10× 10^9^/L and hemoglobin of 92 g/L. He was treated with 500 mg of intravenous methylprednisolone daily and transfused with 4 units of platelets, which increased his platelet count to 36 × 10^9^/L. Upon admission, the patient’s condition rapidly deteriorated. By the third day, he exhibited left-sided mouth deviation, speech difficulties, intermittent agitation, transient right limb weakness with numbness, urinary and fecal incontinence, and disturbances in consciousness. Head CT and CTA scans were normal. Given his underlying SLE and LN, we initially evaluated lupus activity. The results indicated normal complement levels (C3 0.923 g/L; C4 0.253 g/L), a positive anti-ANA (<1:512), negative anti-ds-DNA (<1:10), negative anti-Sm, and negative antiphospholipid antibodies, thereby excluding the possibility of immune thrombocytopenia resulting from increased SLE activity. However, plasma ADAMTS-13 activity was completely absent (0%) without any ADAMTS-13 inhibitors. Clinically, this was considered COVID-19-induced TTP.

**Figure 1.**
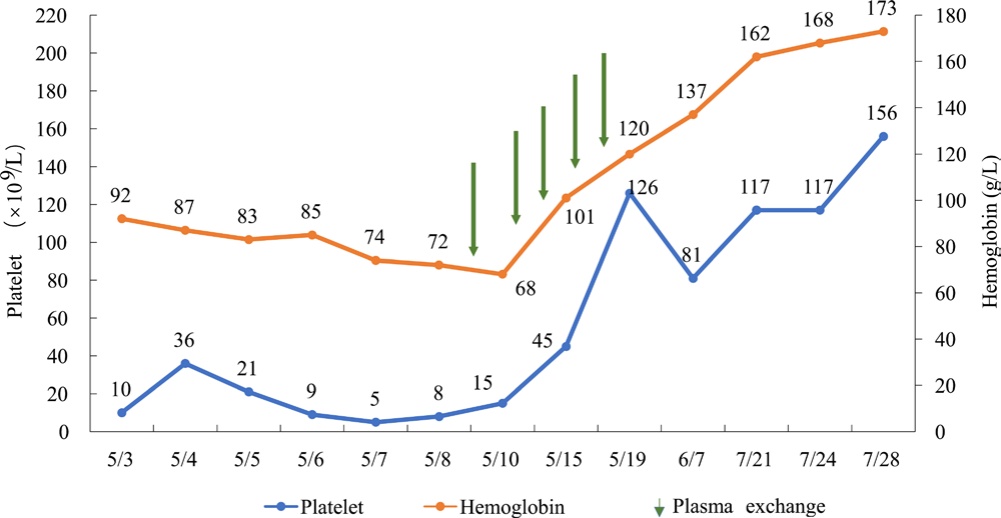
Changes in platelet and hemoglobin levels during the course of illness in this patient in 2023.

For treatment, in addition to the initial platelet transfusions, we administered 500 mg methylprednisolone pulses and conducted 5 sessions of PE after confirming the diagnosis. The patient’s consciousness quickly improved, hematologic damage ameliorated, platelet count increased to 45 × 10^9^/L, anemia improved with hemoglobin reaching 101 g/L, and reexamination showed plasma ADAMTS-13 activity level at 88.79% without any ADAMTS-13 inhibitors. Subsequently, the patient was administered rituximab 600 mg per dose, twice at half-month intervals, and belimumab 720 mg every half-month for 3 doses, followed by 720 mg monthly. Additionally, methylprednisolone combined with tacrolimus therapy was continued. The patient’s hematologic damage gradually recovered, with platelet count increasing to 156 × 10^9^/L and hemoglobin to 173 g/L (Fig. 1).

**Figure 2.**
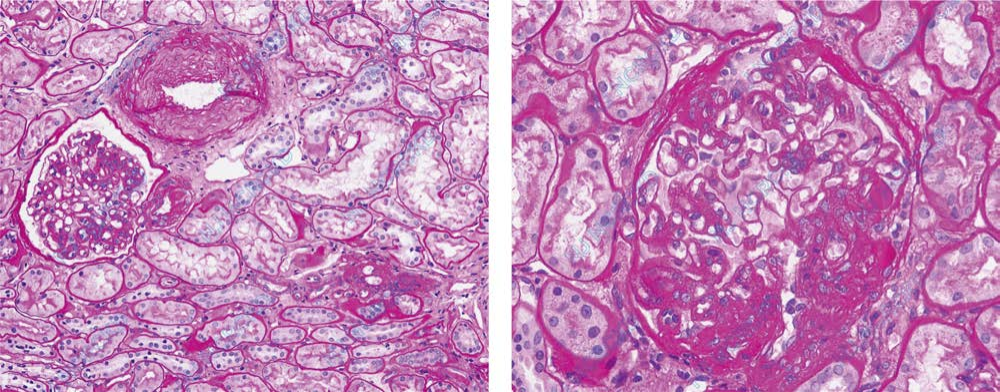
Light microscopy showing increased mesangial cells and matrix, well-opened capillary loops, segmental peripheral loops adhering to the capsule wall, and segmental thickening and layering of the capsule wall (periodic acid–Schiff staining × 400).

After the patient’s hematologic and central nervous system injuries subsided, we conducted a repeat renal biopsy in July 2023 to further assess kidney damage. The light microscopy examination of the biopsy specimen, which contained 49 glomeruli, revealed no global sclerosis. There was an increase in mesangial cells and matrix, along with segmental peripheral loop adhesion to the capsule wall (AI3, CI3). However, the capillary loops remained well open, indicating that TTP had not yet impacted the glomeruli (Fig. 2).

Given the patient’s renal pathology indicating minor proliferative lesions, tacrolimus was discontinued. The patient continued treatment with methylprednisolone combined with mycophenolate mofetil, along with belimumab 720 mg per dose per month.

**Figure 3.**
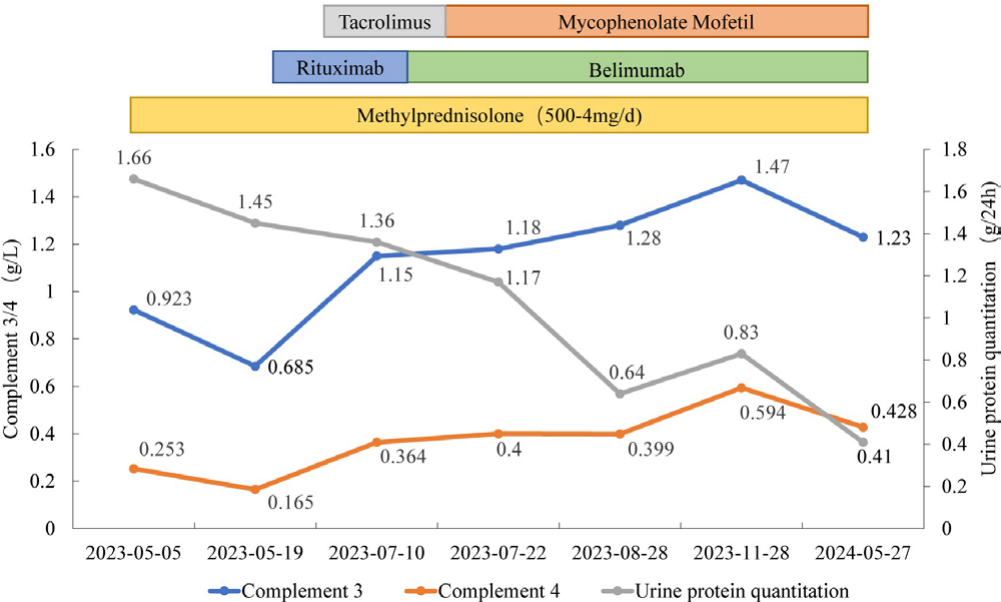
Changes in complement 3/4, urinary protein quantification, and medication use in this patient after onset.

After 1 year of follow-up, the patient’s LN was in remission, with an SLEDAI-2K score of 0, indicating a low disease activity state. Laboratory studies at this time showed a urinary protein quantification of 0.41 g/24h, a urine red blood cell count of 6.0/μL, an albumin level of 47.3 g/L, and a serum creatinine level of 1.05 mg/dL. C3 and C4 levels were 1.23 g/L and 0.428 g/L, respectively, and the anti-ds-DNA antibody was within the normal range (Fig. 3).

## 3. Discussion

The classic clinical manifestations of TTP include the pentad of MAHA, thrombocytopenia, neuropsychiatric symptoms, fever, and renal failure.^[1]^ The pathogenesis of TTP involves endothelial dysfunction caused by autoantibodies that inhibit the metalloprotease ADAMTS-13, which cleaves von Willebrand factor (VWF). Deficiency of this protease results in unusually large VWF (UL-VWF) binding spontaneously with platelets, leading to microvascular thrombosis, microangiopathic hemolysis, and subsequent ischemia, hypoxia, and dysfunction of the affected organs.^[2]^ The clinical presentation of this patient included severe thrombocytopenia with anemia, central nervous system injury, and recurrence of nephropathy. ADAMTS-13 activity testing revealed a complete deficiency, leading to a clinical diagnosis of TTP.

This patient had underlying SLE and LN, and was also infected with the SARS-CoV-2, complicating the differentiation of the cause of TTP. Reviewing the patient’s pathogenetic process, clinical feature, laboratory parameters, and treatment outcomes, we speculate that it is more likely related to SARS-CoV-2. Disease course: the patient’s condition deteriorated suddenly following the COVID-19 infection. COVID-19, caused by the severe acute respiratory syndrome coronavirus 2 (SARS-CoV-2), has been found in studies to possibly trigger auto-inflammatory and autoimmune diseases such as TTP.^[3]^ Chaudhary et al documented 11 cases of TTP induced by COVID-19, with diagnoses generally occurring around 10 days (SD 5.8) after the onset of COVID-19. The mean age was 48.2 years (SD 15.1), dyspnea was the most common symptom (36.6%), and 27.3% of cases exhibited neurological symptoms.^[1]^ In this article, the patient presented with fever, generalized weakness, respiratory symptoms, and thrombocytopenia, and was diagnosed with COVID-19 via nasopharyngeal PCR after hospitalization. Despite a 15-year history of SLE and LN, the patient had been in continuous remission with regular methylprednisolones and immunosuppressive therapy, making SLE-induced TTP less likely. Therefore, the temporal relationship and clinical presentation suggest that the TTP is more likely related to the recent SARS-CoV-2 infection. Clinical and laboratory findings: previous studies have indicated that SLE complicated by TTP is associated with significantly elevated ds-DNA antibodies and more pronounced hypocomplementemia.^[4]^ In this patient, immunological indicators were not significantly active, with positive ANA (1:512), negative anti-ds-DNA, and normal complement levels. Additionally, urinary protein increased only to 1.6g/24 h, renal function and blood pressure remained normal, and a repeat renal biopsy showed no signs of thrombotic microangiopathy. These findings do not support a diagnosis of SLE-induced TTP, further suggesting that the TTP is more likely related to the recent SARS-CoV-2 infection rather than an exacerbation of SLE. ADAMTS-13 activity: ADAMTS-13 activity testing is critical for diagnosing TTP. This patient’s ADAMTS-13 activity level was 0%. In the 11 cases reported in the literature, ADAMTS-13 activity levels were < 10%, and 8 cases (72%) had elevated ADAMTS-13 inhibitors or antibody levels.^[1]^ In COVID-19 patients, ADAMTS-13 activity levels are typically reduced, creating a pro-thrombotic environment. This reduction may be related to increased pro-coagulant factors (such as factor VIII, vWF, and fibrinogen) and endothelial damage caused by cytokines induced by COVID-19.^[5]^ The decrease in ADAMTS-13 activity levels is associated with the severity and prognosis of the viral infection and may increase mortality.^[6]^ ADAMTS-13 changes are more complex in SLE complicated by TTP. Studies have shown that ADAMTS-13 activity is also low in SLE patients with positive anti-ds-DNA. This phenomenon may result from an imbalance in the regulatory mechanism between ADAMTS-13 and vWF.^[4]^ During the course of SLE, inflammation, autoimmune responses, and vascular damage lead to a significant increase in vWF multimers, causing an initial regulatory increase and subsequent significant depletion of ADAMTS-13. However, studies have found no cases of severe ADAMTS-13 deficiency (<5%) in SLE patients, with the lowest recorded being 13%, and a negative correlation with significantly increased vWF levels.^[7]^ Therefore, the patient’s ADAMTS-13 activity test results do not match previously reported SLE-TTP cases, further suggesting that the TTP in this patient is more likely related to the recent SARS-CoV-2 infection rather than an exacerbation of SLE. Other antibodies: the occurrence of SLE-TTP is related to the presence of various antibodies such as anti-endothelial cell antibodies, antiplatelet antibodies, and anti-ADAMTS-13 antibodies. This patient’s antiplatelet antibody IgG (PAIgG) and platelet-specific autoantibodies were negative. Antiphospholipid antibodies are also an important mechanism in SLE-TTP, with 30% of SLE-TTP patients having positive antiphospholipid antibodies.^[8]^ This patient’s antiphospholipid antibody test result was negative. Treatment outcome: the patient’s condition improved rapidly after treatment, and ADAMTS-13 activity quickly returned to normal levels. This rapid improvement was partly due to active methylprednisolone pulse therapy and PE treatment. More importantly, it was likely due to recovery from the COVID-19 infection, supporting the association of the patient’s TTP with the SARS-CoV-2 virus. SLE complicated by TTP usually presents a more severe condition. Although PE significantly improves TTP prognosis, with survival rates exceeding 80%, TTP associated with SLE tends to be more severe and fatal, with mortality rates ranging from 34.1% to 62.5%, and a poorer response to treatment.^[9]^ Therefore, combining the patient’s clinical course, laboratory parameters, and treatment outcomes, the evidence does not support SLE-induced TTP. Additionally, we performed bone marrow tests that showed only decreased megakaryocyte proliferation, elevated reticulated platelet levels, a negative Coombs test, a negative Ham test, and a negative paroxysmal nocturnal hemoglobinuria (PNH) antigen test, ruling out intrinsic bone marrow diseases, PNH, and immune thrombocytopenia. In summary, the cause of TTP in this patient is considered to be the COVID-19 infection.

Current research suggests that the primary treatment for TTP caused by COVID-19 infection should focus on the underlying cause, controlling the COVID-19 infection, followed by specific treatment for TTP. The 2020 ISTH guidelines for TTP treatment suggest that adding methylprednisolone therapy to PE reduces mortality compared to using PE alone. However, the specific type and dosage of methylprednisolones remain unclear. In addition to PE and methylprednisolones, the use of rituximab and caplacizumab is also recommended.^[10]^ Rituximab is a monoclonal antibody targeting CD20 on B cells. Administering it immediately after PE can prevent reduced drug exposure caused by exchange and prolong the drug response rate.^[11]^ However, the American Society of Hematology considers rituximab impact on antibody production and its association with viral reactivation, which may increase the risk of primary viral infections. It is recommended to use this drug cautiously in COVID-TTP patients. Caplacizumab is a novel drug that can block the interaction between UL-VWF and platelets, preventing microvascular thrombosis and serving as an adjunct treatment to PE and immunosuppressants. Current research suggests that the main adverse effect of caplacizumab is mucocutaneous bleeding, and it may delay the recovery of severe ADAMTS-13 deficiency.^[12]^ This drug is not yet approved in China and was therefore not used in this case.

## 4. Conclusion

This article reports the first case of TTP complicating SLE following COVID-19 infection. Given the patient’s underlying SLE and the sudden viral infection, the differential diagnosis and etiological treatment of TTP are particularly challenging. In this case, the patient was successfully treated with PE, followed by a combination of biologics and immunosuppressants. The long-term prognosis remains to be determined through prolonged follow-up.

## Author contributions

**Data curation:** Ou Gao.

**Funding acquisition:** Honglang Xie.

**Investigation:** Ou Gao.

**Methodology:** Yinghua Chen.

**Project administration:** Honglang Xie.

**Resources:** Honglang Xie.

**Software:** Yinghua Chen.

**Writing – review & editing:** Honglang Xie, Yinghua Chen.

**Writing – original draft:** Ou Gao.
